# Effects of sources of social support and resilience on the mental health of different age groups during the COVID-19 pandemic

**DOI:** 10.1186/s12888-020-03012-1

**Published:** 2021-01-07

**Authors:** Fugui Li, Sihui Luo, Weiqi Mu, Yanmei Li, Liyuan Ye, Xueying Zheng, Bing Xu, Yu Ding, Ping Ling, Mingjie Zhou, Xuefeng Chen

**Affiliations:** 1 CAS Key Laboratory of Mental Health, Institute of Psychology, Chinese Academy of Sciences, 16 Lincui Road, Chaoyang District, Beijing, 100101 China; 2 Department of Psychology, University of Chinese Academy of Sciences, Beijing, 100049 China; 3 The First Affiliated Hospital of USTC, Division of Life Sciences and Medicine, University of Science and Technology of China, Hefei, 230001 China; 4 Institute of Public Health, University of Science and Technology of China, Hefei, 230026 China; 5 CAS Key Laboratory of Behavioral Science, Institute of Psychology, Chinese Academy of Sciences, 16 Lincui Road, Chaoyang District, Beijing, 100101 China

**Keywords:** COVID-19, Social support, Resilience, Mental health, Age difference

## Abstract

**Background:**

A pandemic is a very stressful event, especially for highly vulnerable people (e.g., older adults). The purpose of the current study was to investigate the main and interactive relationships of social support and resilience on individual mental health during the COVID-19 pandemic across three age groups: emerging adults, adults, and older adults.

**Methods:**

A survey was conducted with 23,192 participants aged 18–85. Respondents completed a questionnaire, including items on the COVID-19-related support they perceived from different sources, the abbreviated version of the Connor-Davidson Resilience Scale, and the Mental Health Inventory.

**Results:**

Latent profile analysis identified five profiles of social support, and the patterns of potential profiles were similar in all groups. However, category distribution in the five profiles was significantly different among the age groups. Furthermore, analysis using the BCH command showed significant differences in mental health among these profiles. Lastly, interactive analyses indicated resilience had a positive relationship with mental health, and social support served as a buffer against the negative impact of low resilience on mental health.

**Conclusions:**

This study provides quantitative evidence for socioemotional selectivity theory (SST) and enables several practical implications for helping different age groups protecting mental health during pandemic.

## Background

The COVID-19 pandemic, which the World Health Organization called a public health emergency of international concern, has received global attention due to its rapid transmission and for causing varying degrees of illness. Contact tracing, case isolating, and quarantining have proven to be effective ways to control the outbreak of infectious disease [1], and many countries worldwide have adopted such measures. Therefore, unlike other crises, the COVID-19 pandemic has likely brought many changes to how individuals live, along with uncertainty, altered daily routines, financial pressures, and social isolation. Hence, the pandemic may also create heavy psychological and emotional burdens for the general population. A pandemic is a very stressful event, and while it is reasonable to feel stress and anxiety, it is also prevalent for people to display high resilience during crises. Moreover, as people face an onslaught of stressors related to the disruptions in their lives caused by the pandemic, they should rely on each other for connection and coping strategies to ease the weight of the public health crisis on their mental health. Therefore, understanding individual mental health outcomes and related protective factors (e.g., social support, psychological resilience) can help provide better targeted suggestions and assistance for different people.

As the scientific community and clinicians immediately identified, the mortality risk of COVID-19 is age-related, with the death toll skewing strongly toward older adults. According to the latest data reported in Nature Medicine, compared to those aged 30–59 years, those aged below 30 and above 59 years were 0.6 and 5.1 times more likely to die after developing symptoms, respectively [2]. Therefore, older adults may experience more stress than other age groups during this pandemic. Thus, it is essential to explore how different age groups behave in this pandemic. Hence, in this study, we aimed to examine the relationships between social support, resilience, and mental health in different age groups during the COVID-19 pandemic.

## Literature review

### Social support and mental health

Social support has been described as “support accessible to an individual through social ties to other individuals, groups, and the larger community” [3]. Although social support can be measured in many ways, perceived social support is the most commonly measured index of social support [4], given its ease of measurement and evidence that it is a better predictor of mental health than other measures [5, 6]. A wealth of research has demonstrated the protective effect of perceived social support on mental health in stressful situations [7–9]. Besides, received social support (a receipt of supportive behaviors) is also an important sub-construct of social support [10]. Although the distinction between perceived social support and received social support exists, from the perspective of the stress and coping on social support [11], it is believed that the relationship between perceived and received social support should be relatively high, especially when the support demand matches the type of support provided [12]. Similarly, some authors suggest that perceived support can be assessed through the recall of supportive behaviors provided [13]. We believed that during the COVID-19 pandemic, an individual’s need for support matches the type of support provided, therefore, we measured social support individuals received to reflect the social support individual perceived.

It has been suggested that social support and social support resources should be viewed as related but distinct concepts [14]. Perceived support represents one’s subjective perceptions of the extent to which social network members are available to provide social support [15]. Several studies [16] have concluded that perceived quality of support is more strongly associated with mental health than with the actual structure of personal networks. Generally, perceived social support can come from a variety of sources, including, but not limited to, family, friends, romantic partners, pets, community ties, and coworkers. A wealth of research has demonstrated that different sources of social support have different influences on mental health in youth [17–19]. Specifically, social support from family, but not from friends was related to posttraumatic stress disorder and depression symptoms [17]. And peer social support had a stronger protective effect against psychological distress than did family social support in another study [19]. Sources of support can be natural (e.g., family and friends) or more formal (e.g., mental health specialists or community organizations), and it is necessary to consider the different sources of social support when exploring the relationship between social support and mental health [20].

The different sources of social support will co-exist within persons, and combine to form different configural profiles. The traditional method, such as variable-centered methods, may overlook differences in social support among heterogeneous groups of individuals. Although there are many papers on sources of social support that have relied on the variable-centered methods. New methods (e.g. Latent profile analysis, LPA) have received growing interest in the typologies of social support networks in recent years (e.g. [21, 22]). What’s more, although based on slightly different concepts, some researchers have used cluster analysis to study social network typologies for a long time (e.g. [23]). More importantly, the existing papers demonstrated different support profiles would be differently associated with mental health outcomes [21, 22, 24]. Specifically, Burholt et al. [21] adopted LPA and found that the four social support profiles differ in their relationships to wellbeing in older migrants. The migrants with family-oriented profiles will experience lower levels of loneliness. Lau et al. [22] found that as compared with participants in the family-dependent social support profile, older adults in the locally integrated social support profile were negatively associated with dementia. McConnell et al. [24] adopted cluster analysis and explored how the combinations of different sources of support impact mental health among lesbian, gay, bisexual, and transgender youth. Researchers found that social support profiles showed different patterns of association with mental health outcomes. Individuals with the high support profile being the least lonely; youth low in all sources of social support are the most vulnerable for hopelessness and anxiety; social support from peers and significant others—even in the absence of family support—play an important protective role in hopelessness and anxiety [24].

Compared to the traditional clustering method, LPA is more reasonable to test the results of individual clustering by directly estimating the membership probability from the model ([25, 26]). Specifically, LPA is a person-centered method that identifies subgroups of individuals who share common social support patterns [27]. Besides, the outcome variables can be incorporated into the LPA model to build a regression mixture model to verify the relationship between profiles and outcomes. Hence, we conducted an LPA to explore the profiles of sources of social support and adopted the regression mixture model (BCH method) to explore the relationship between social support profiles and mental health in this study.

Unlike other crises or disasters (e.g., earthquake, economic crisis), a wider range of people will face a series of stressful events due to the pandemic itself and the related quarantine measures; therefore, the support provided both within the micro context (e.g., spouse, family, friends) and macro context (e.g., community, institutions/organizations, or even society as a whole) is vital to help ease psychological burdens. Accordingly, along with family support, it is also essential to consider the role of other sources of social support in mental health within a broader context. Furthermore, it is important to acknowledge individuals may experience different relative levels of these forms of support (e.g., high levels of support from community or even society as a whole, but low levels of family support) during the COVID-19 pandemic. However, little is known about how combinations of different sources of social support impact mental health during this period. Since individuals will face a series of stressful events during the COVID-19 pandemic, we infer that people with a high level of social support profile will experience a higher level of mental health than those with moderate or low social support profiles. Therefore, our first hypothesis is as follows:

- H1a: Sources of support, including family, friends or small groups, communities, organizations/institutions, and society as a whole, positively associate with an individual’s mental health during the COVID-19 pandemic.
- H1b: Individuals within different social support profiles showed different relationships with mental health during the COVID-19 pandemic. People with a high level of social support profile will experience a higher level of mental health than those with moderate or low social support profiles.

### Resilience and mental health during a crisis

We can often observe that, although many people may suffer from difficulties or crises, mental health outcomes still differ among individuals, and psychological resilience is assumed to play an important role in explaining such variance. Psychological resilience can be considered as either a trait or a process/outcome [28]. As a process, researchers referred to it as a dynamic process encompassing positive adaptation within the context of significant adversity [29]. As a trait, resilience represents a constellation of characteristics that enable individuals to adapt to the circumstances they encounter [30] and resilience has a positive impact on individual mental health [31]. A meta-analysis suggested that resilience may play a role in protecting mental health, accelerating recovery, and mitigating the negative effects of a crisis [32]. For instance, Tugade and Fredrickson [33] describe resilience as a quick and effective recovery after stress, while Patel and Goodman [34] conceptualize resilience as preserving mental health in the face of adversity. Therefore, as a trait, psychological resilience is a key resource in helping to prevent the negative impact of a crisis. And our second hypothesis is as follows:

- H2: Psychological resilience positively associates with an individual’s mental health during the COVID-19 pandemic.

### Interaction effect of social support and resilience on mental health

As previously noted, sources of support and psychological resilience are two resources that protect individual mental health in stressful situations. Research shows that social support is key to resilience when it is considered to be a process/outcome (e.g., [35, 36]). However, when resilience is considered to be a trait, we cannot help asking, since there are individual differences in social support and psychological resilience, could abundance in perceived social support compensate for lack of psychological resilience? One study found that sources of social support (family, friends, and others) significantly moderated the relationship between resilience and subjective well-being in college students [8], while another study demonstrated that the relationship between resilience and psychological distress was not moderated by social support in patients diagnosed with cancer [37]. According to the stress-buffering hypothesis, researchers claimed that social support acted as a buffer to alleviate the negative influence of stress on well-being [38]. Scholars found that family support played a unique role in buffering the negative effects of stress by considering the different sources of support [39]. What’s more, Anschuetz [40] found that social support served to attenuate the negative effects of various trait vulnerabilities (such as neuroticism and introversion). Based on the stress-buffering hypothesis, we claim that social support from different sources can also buffer the negative effect of low levels of resilience (a trait vulnerability) on mental health, especially in a brand-new context, such as the COVID-19 pandemic. Since we adopt LPA to identify the profiles of the sources of social support, we extend the compensation hypothesis to social support profiles. Hence, our third hypothesis is as follows:

- H3: Social support profiles moderate the relationship between resilience and mental health during the COVID-19 pandemic. People with a high level of social support profile will buffer the negative effect of low levels of resilience on mental health during the COVID-19 pandemic.

### Sources of social support, psychological resilience, and mental health, varied across life stages

As a global public health emergency, the COVID-19 pandemic has caused great suffering to all people living in affected areas. When considering individual mental health as well as protective factors during the COVID-19 pandemic, age is a significant and unavoidable issue. First, many studies have shown that emotional well-being often increases across the entire adult life stage; thus, with age, people’s emotions and well-being tend to remain stable or even improve. Researchers refer to this phenomenon as the “paradox of aging” [41]. Moreover, according to the latest data reported in Nature Medicine, compared to those aged 30–59 years, those aged below 30 and above 59 years were 0.6 and 5.1 times more likely to die, respectively, after developing symptoms of COVID-19 [2] . Hence, older adults may experience more stress than other age groups. Therefore, the question remains as to whether the paradox of aging still exists within the context of the COVID-19 pandemic. However, according to the paradox of aging [41], we hypothesize that:

- H4: The paradox of aging still exists in individuals even during the COVID-19 pandemic, which means the levels of mental health in older adults are higher than others.

Second, according to socioemotional selectivity theory (SST), as individuals become more aware of time limitations in life as they age, they are more likely to spend time with emotionally close relationships than young people are [42–45]. SST also suggests that, with age, individuals narrow the size of their social network and focus on a smaller circle of friends and relatives [42–45]. Researchers found that older adults have a greater proportion of close social partners and fewer peripheral partners in their networks compared to younger adults [46, 47]. Within social support research, Potts [48] found that the older adults received different levels of social support from within and outside the retirement community because of the different levels of social network quantity between the within and outside the retirement community. Thus, for individuals of different ages, in terms of differences in their social networks, it is expected that the levels of sources of social support they received are different. Furthermore, a meta-analysis that systematically reviewed the characteristics of social support (types and sources) associated with protection from depression across life stages (e.g., childhood and adolescence, adulthood, older age), found that sources of support varied across life stages, with parental support being the most important among children and adolescents, whereas adults and older adults relied more on spouses, followed by family and then friends [49]. Hence, it is worth exploring whether when everyone is under a situation in which people cannot avoid being involved, such as the COVID-19 pandemic, if there are age differences in perceived sources of social support. Therefore, according to SST, we hypothesize that:

- H5: Older adults have higher levels of family support than others, which means the distribution of older groups in high family support profile is higher than that of other groups.

Third, research suggests that psychological resilience may vary according to age [50]. A survey based on a large cross-sectional Japanese sample demonstrated that psychological resilience could increase from the young adult stage to the older adult stage [51]. Furthermore, research showed that cancer patients with higher resilience, particularly older patients, experienced lower psychological distress, indicating that the relationship between resilience and psychological distress is moderated by age [37]. Research also demonstrated that age moderated the relationship between sources of social support and mental health, indicating that family social support is associated with lower symptoms of posttraumatic stress disorder and depression in participants aged 16–19 years, while friend social support is associated with lower symptoms for participants over 20 years old [17]. The possible role of age was examined in these analyses, especially the age differences in associations between resilience and mental health [37], and the moderating role of age between sources of support and mental health [17]. As previously noted, according to the stress-buffering hypothesis [38], people with a higher level of social support profile may buffer the negative effect of low levels of resilience on mental health. Few studies have examined whether the buffering effects of social support profiles vary by age, but the role of age has been associated with the variables of interest. What’s more, according to SST, older groups will have a greater proportion of high family-based support profile than the other age groups. Therefore, we hypothesize that the buffering effect between the high family-based support profile and resilience is greater in older adults:

- H6: The compensation effects of social support profiles show different patterns across age groups during the COVID-19 pandemic. Specifically, the buffering effect between the high family-based support profile and resilience is greater in older adults.

## Methods

### Participants

The study protocol was approved by the institutional review board: Ethics Committee of Institute of Psychology, Chinese Academy of Sciences (reference number: H20016) and all respondents signed informed consent before participation. Data were collected from 23,192 participants via the First Affiliated Hospital of the University of Science and Technology of China online data platform, mostly from the Anhui Province (98.97%), from March 25 to April 1, 2020. Respondents’ ages ranged from 18 to 85 (*M* = 41.58, *SD* = 14.63), and 7437 (32.1%) were male. Given that the data reported in Nature Medicine, the fatality rate is different among different age groups (below 30, 30–59, above 60) [2], the population was stratified into different age groups in this study. Furthermore, emerging adult, which a focus on ages 18–25, differs from adolescence and young adult according to Arnett’s study [52]. We used age as a categorical variable rather than a continuous variable and the three groups were divided by 25 and 60 years (18–25, 25–59, above 60). The three age groups were emerging adults (2045 participants, 31.10% male, range = 18–25), adults (18,159 participants, 29.50% male, range = 26–59), and older adults (2988 participants, 48.5% male, range = 60–85).

### Measurements

#### COVID-19-related support

Based on the concept of “sources of support” [49], we developed a checklist measure, with a total of five questions. This checklist asked participants about the degree of support and assistance they received from different sources, including family, friends or small groups, communities, organizations or institutions, and society as a whole, during the COVID-19 pandemic. The extent of support received was measured on a 5-point scale, ranging from 1 (none) to 5 (a great many). Cronbach’s α was 0.90 in the total sample.

#### Resilience

We used the abbreviated version of the Connor-Davidson Resilience Scale (CD-RISC) [53], which was designed by Vaishnavi et al. to measure respondents’ levels of resilience. It comprises two items (“Able to adapt to change”, “Tend to bounce back after illness or hardship”), and both are rated on a 5-point Likert-type scale, ranging from 1 (not at all) to 5 (extremely). The higher the total score, the higher the level of psychological resilience. Cronbach’s α was 0.79 in the total sample.

#### Mental health

The Mental Health Inventory (MHI-5) [54] designed by Berwick et al. was used to measure respondents’ mental health during the past month. It comprises five items, each rated on a 6-point Likert-type scale, ranging from 1 (almost all the time) to 6 (never). The higher the total score, the higher the level of mental health. Cronbach’s α was 0.79 in the total sample.

#### Control variable

As previous research highlighted that there were gender differences in social support, resilience and mental health [55, 56]. Besides, people with underlying diseases were at greater risk of developing into severe diseases during the COVID-19 pandemic [57]. Gender and disease history which including lung disease, hypertension, kidney disease, and other primary diseases were controlled in this study.

### Analytic strategy

SPSS version 21.0 was used for descriptive statistics, one-way analysis of variance (ANOVA), and chi-square tests.

LPA with a continuous dependent variable was performed using Mplus version 8.0, and the algorithm adopted was the BCH method [58]. First, LPA was used to identify the number and nature of classes of COVID-19-related support in all samples, with the scores of the five items that the support and assistance participants received from different sources as extrinsic variables, respectively. A series of models with progressively increasing numbers of classes from 2 to 6 were estimated and compared to determine the optimal model solution based on lower Akaike information criterion (AIC) values, lower Bayesian information criterion (BIC) values, significant Lo–Mendell–Rubin likelihood ratio test (LMR-LRT), bootstrap likelihood ratio test (BLRT) *p*-values, considerable class sizes (at least 5% of the sample), and higher entropy values [59]. To be specific, we based on the rules that the model with the lowest BIC and AIC index offered the best-fit indicator, and the significant test of LMR and BLRT showed that the k + 1 profile was superior to the k-profile. Moreover, we take the entropy into account, the higher the entropy, the more accurate the classification. More importantly, we followed the advice that if the additional profile adds a qualitatively new profile to the prior solution, the new profile might be retained. On the contrary, if an additional profile is only minor level differences in all profile variables compared to the prior solution, the new profile might not be retained due to reasons of parsimony [25, 60]. Second, for the outcomes, we used the BCH command, in which analyses reflect whether one profile is statistically significantly different from other profiles per criterion. Meanwhile, testing mean differences across the profile groups to mental health outcomes is also a validation of the classification results according to Daniel [26]. Third, the interaction between social support and resilience on mental health across different groups during the COVID-19 pandemic was assessed using the PROCESS version 3.4 macro of SPSS version 21.0, developed by Hayes [61]. In this approach, moderating effects were tested when 95% bias-corrected bootstrap confidence intervals based on 5000 samples did not contain zero.

## Results

Means, standard deviations, and correlations are presented in Table 1. The results showed that sources of support (including family, friends or small groups, communities, organizations/institutions, and society as a whole) and resilience positively associated with mental health. Thus, hypothesis 1a and hypothesis 2 were supported. To examine differences among the three age groups for all variables, we conducted a one-way ANOVA for the total sample. For COVID-19-related support, the group differences were significant in the five dimensions of family, friends or small groups, communities, organizations or institutions, and society as a whole (*F*_*family*_ [2, 23,189] = 6.93, *p* = .001, partial *η*^2^ = .001; *F*_*friends*_ [2, 23,189] = 24.08, *p* < .001, partial *η*^2^ = .002; *F*_*communities*_ [2, 23,189] = 8.94, *p* < .001, partial *η*^2^ = .001; *F*_*organizations*_ [2, 23,189] = 30.14, *p* < .001, partial *η*^2^ = .003; *F*_*society*_ [2, 23,189] = 26.91, *p* < .001, partial *η*^2^ = .002). Post-hoc analysis revealed that emerging adults reported higher support (except for the family dimension) than adults, and adults reported higher support than older adults (*p*_*s*_ < .01). As for the family dimension, adults reported the lowest support, and there was no significant difference between emerging adults and older adults (*p* = .202). For resilience and mental health, the group differences were significant (*F*_*resilience*_ [2, 23,189] = 3.84, *p* = .021, partial *η*^2^ < .001; *F*_*mental health*_ [2, 23,189] = 5.90, *p* = .003, partial *η*^2^ = .001). Post-hoc analysis revealed that adults and older adults reported higher resilience than emerging adults (*p* = .006, *p* = .024), and there was no significant difference between adults and older adults (*p* = .974). Further, post-hoc analysis revealed that older adults reported higher mental health than emerging adults and adults (*p* = .045, *p* = .001), and there was no significant difference between emerging adults and adults (*p* = .659). In line with hypothesis 4, the results revealed that older adults owned higher levels of mental health than others. Therefore, hypothesis 4 was supported.

**Table 1:** Means, standard deviations and correlations amongst study variables

		***Rang***	***M***	***SD***	1	2	3	4	5	6	7
Total sample (*N* = 23,192)	1family	1–5	3.56	1.08	1						
	2friends	1–5	3.02	1.14	.61**	1					
	3communities	1–5	2.96	1.16	.51**	.67**	1				
	4organizations	1–5	2.89	1.20	.46**	.65**	.82**	1			
	5society	1–5	3.13	1.16	.50**	.61**	.75**	.77**	1		
	6resilience	1–5	3.76	0.71	.24**	.21**	.20**	.18**	.20**	1	
	7mental health	1–6	5.01	0.77	.18**	.13**	.12**	.10**	.14**	.40**	1
Emerging sample (*n* = 2045)	1family	1–5	3.63	1.04	1						
	2friends	1–5	3.13	1.14	.56**	1					
	3communities	1–5	3.03	1.15	.47**	.64**	1				
	4organizations	1–5	3.01	1.17	.45**	.62**	.82**	1			
	5society	1–5	3.25	1.15	.45**	.57**	.73**	.77**	1		
	6resilience	1–5	3.72	0.70	.30**	.25**	.25**	.25**	.22**	1	
	7mental health	1–6	5.01	0.76	.26**	.19**	.19**	.18**	.18**	.38**	1
Adult sample (*n* = 18,159)	1family	1–5	3.55	1.08	1.00						
	2friends	1–5	3.03	1.15	.62**	1.00					
	3communities	1–5	2.97	1.16	.52**	.67**	1.00				
	4organizations	1–5	2.90	1.21	.47**	.65**	.82**	1.00			
	5society	1–5	3.14	1.16	.51**	.61**	.75**	.77**	1.00		
	6resilience	1–5	3.77	0.71	.24**	.21**	.20**	.18**	.20**	1.00	
	7mental health	1–6	5.00	0.78	.18**	.13**	.11**	.09**	.14**	.40**	1.00
Older sample (*n* = 2988)	1family	1–5	3.59	1.07	1.00						
	2friends	1–5	2.91	1.12	.55**	1.00					
	3communities	1–5	2.89	1.14	.47**	.67**	1.00				
	4organizations	1–5	2.76	1.18	.42**	.64**	.82**	1.00			
	5society	1–5	3.01	1.13	.47**	.61**	.77**	.80**	1.00		
	6resilience	1–5	3.77	0.68	.20**	.17**	.16**	.16**	.17**	1.00	
	7mental health	1–6	5.05	0.71	.13**	.10**	.12**	.12**	.14**	.40**	1.00

Note: **p < .01

We did LPA in the total sample as well as in each age group. The model fit indices are shown in Table 2. AIC, BIC, and aBIC values continued to decline from two-profile to six-profile in these samples; the 5-class solution was found to have the best data fit. Although the 4-class solution had a higher entropy value compared to the 5-class solution, the 5-class solution highlighted a “relatively high family support, low other social supports group” (class 2), which could reveal the vibrant pattern of social support resources and better highlight the advantages of LPA [25, 60]. Furthermore, considering theoretical parsimony and the smallest class proportion, we selected the 5-profile solution instead of the 6-class solution in the total sample and subsamples [25, 60].

**Table 2:** Fit statistics for latent profile analyses

Samples	Models	AIC	BIC	aBIC	Entropy	LMR LRT	BLRT	Smallest Class Proportion (%)
Total sample (*N* = 23,192)	2- profile	315,765.06	315,893.88	315,843.03	0.88	<.001	<.001	35.5
	3- profile	294,089.31	294,266.44	294,196.53	0.89	<.001	<.001	26.8
	4- profile	286,551.52	286,776.96	286,687.98	0.90	<.001	<.001	10.0
	5- profile	280,090.42	280,364.17	280,256.12	0.90	<.001	<.001	9.9
	6- profile	276,683.56	277,005.62	276,878.50	0.90	<.001	<.001	5.1
Emerging sample (*n* = 2045)	2- profile	27,715.47	27,805.44	27,754.61	0.89	<.001	<.001	34.2
	3- profile	25,810.53	25,934.24	25,864.35	0.90	<.001	<.001	26.8
	4- profile	25,032.71	25,190.16	25,101.20	0.92	<.001	<.001	11.6
	5- profile	24,653.00	24,844.19	24,736.17	0.90	<.001	<.001	11.5
	6- profile	24,378.326	24,603.252	24,476.169	0.91	0.007	<.001	5.2
Adult sample (*n* = 18,159)	2- profile	247,466.10	247,591.01	247,540.17	0.88	<.001	<.001	35.7
	3- profile	230,816.27	230,988.02	230,918.11	0.89	<.001	<.001	27.3
	4- profile	224,955.50	225,174.10	225,085.11	0.90	<.001	<.001	10.2
	5- profile	219,800.07	220,065.51	219,957.46	0.89	<.001	<.001	10.1
	6- profile	217,019.74	217,332.01	217,204.89	0.90	<.001	<.001	5.2
Older sample (*n* = 2988)	2- profile	40,363.46	40,459.50	40,408.66	0.84	<.001	<.001	36.5
	3- profile	37,259.74	37,391.80	37,321.89	0.90	<.001	<.001	24.1
	4- profile	36,433.68	36,601.74	36,512.78	0.90	<.001	<.001	7.0
	5- profile	35,446.91	35,650.99	35,542.96	0.90	<.001	<.001	7.6
	6- profile	34,896.99	35,137.09	35,009.99	0.91	<.001	<.001	5.8

Note: *AIC* Akaike Information Criterion, *BIC* Bayesian Information Criterion, *aBIC* adjusted BIC, *LMR LRT* Lo-Mendell-Rubin likelihood ratio test, *BLRT* Bootstrap parametric likelihood ratio test

**Fig. 1:**
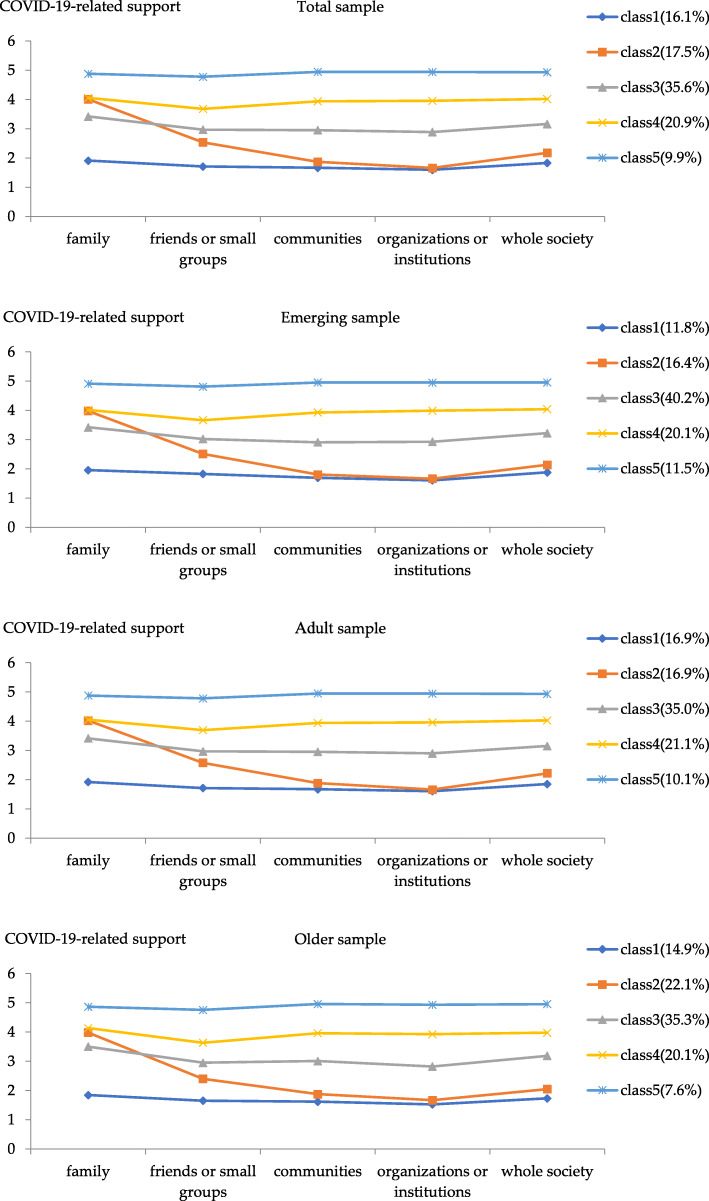
COVID-19-related support’s latent classes in the total sample and the subsamples. Note. class 1 = low social support class, class 2 = predominantly proximal support class, class 3 = predominantly remote support class, class 4 = moderate social support class, class 5 = high social support class

The scores of each COVID-19-related support for the 5-profile model are shown in Fig. 1. Of the five classes, class 1 scored the lowest in all dimensions, and we named class 1 the low social support class. Class 2 scored higher on family dimensions and scored lower on the other dimensions. Therefore, we named class 2 the predominantly proximal support class. Class 3 scored lower on family dimensions and scored higher on the other dimensions. Therefore, we named class 3 the predominantly remote support class. Class 4 scored moderate in all dimensions, and class 5 scored the highest in all dimensions. Therefore, we named class 4 and class 5 the moderate social support class and the high social support class, respectively. From Fig. 1, we can see that the patterns of the potential profiles are very similar in these samples. Also, the descriptions of the classes in the four samples are shown in Table 3 for clarity.

**Table 3:** The descriptions of the classes in four samples

Total sample	family	friends or small groups	communities	organizations or institutions	whole society
class1(16.1%)	1.91	1.71	1.67	1.60	1.83
class2(17.5%)	4.01	2.54	1.87	1.66	2.18
class3(35.6%)	3.42	2.97	2.95	2.89	3.16
class4(20.9%)	4.06	3.68	3.94	3.96	4.02
class5(9.9%)	4.88	4.78	4.95	4.94	4.94
**Emerging sample**					
class1(11.8%)	1.96	1.83	1.69	1.61	1.88
class2(16.4%)	3.98	2.51	1.81	1.66	2.14
class3(40.2%)	3.42	3.02	2.91	2.93	3.22
class4(20.1%)	4.01	3.66	3.93	3.99	4.04
class5(11.5%)	4.91	4.81	4.96	4.95	4.95
**Adult sample**					
class1(16.9%)	1.92	1.71	1.67	1.61	1.85
class2(16.9%)	4.02	2.58	1.88	1.66	2.22
class3(35.0%)	3.41	2.97	2.95	2.90	3.15
class4(21.1%)	4.05	3.69	3.94	3.96	4.03
class5(10.1%)	4.88	4.78	4.95	4.95	4.93
**Older sample**					
class1(14.9%)	1.84	1.65	1.62	1.53	1.73
class2(22.1%)	3.98	2.40	1.87	1.67	2.05
class3(35.3%)	3.50	2.95	3.01	2.82	3.19
class4(20.1%)	4.13	3.63	3.96	3.93	3.98
class5(7.6%)	4.86	4.76	4.96	4.93	4.95

Note. class 1 = low social support class, class 2 = predominantly proximal support class, class 3 = predominantly remote support class, class 4 = moderate social support class, class 5 = high social support class

We conducted a chi-square test to examine the differences in five classes among the three age groups in the total sample. The results showed that there were significant differences among the three groups in five categories (*χ*2(8) = 90.65, *p* < .001). Specifically, the distribution of emerging adults in class 1 (low social support) was significantly lower than that of adults and older adults (11.6% < 16.1% = 15.3%). The distribution of emerging adults and adults in class 2 (predominantly proximal support/ family-based support profile) was significantly lower than that of older adults (17.3% = 17.8% < 22.6%). The distribution of emerging adults in class 3 (predominantly remote support) was considerably higher than that of adults and older adults (39.3% > 35.1% = 34.8%). The distribution of the three groups in class 4 (moderate social support) showed no significant differences. The distribution of emerging adults in class 5 (high social support) was significantly higher than that of adults, and the distribution of adults in class 5 (high social support) was considerably higher than that of older adults (11.3% > 9.8% > 7.4%). Therefore, hypothesis 5 was supported.

To answer the associations between social support profiles and mental health outcome, we conducted an automatic BCH model across the five classes. Results are presented in Table 4, which showed that there were statistically significant differences among the classes for mental health. Specifically, for the total sample and the subsamples, people with a high level of social support profile (class 5) will experience a higher level of mental health than other social support profiles. Therefore, hypothesis 1b was supported. For the emerging adults, there were no statistically significant differences among class 2, class 3, and class 4 for mental health. For the adults, the mental health of class 2 was higher than that of class 3, and there was no statistically significant difference between class 2 and class 4, indicating that family support is more important than other sources of support for adults. For the older adults, there were no statistically significant differences among class 1, class 2, and class 3 for mental health.

**Table 4:** 3-Step results for mental health (BCH)

	class1 (M)	class2(M)	class3(M)	class4(M)	class5(M)	***χ***2	***p*** < .05
Total	4.91	5.04	4.90	5.04	5.44	1011.35***	(1 ≈ 3) < (2 ≈ 4) < 5
Emerging	4.74	4.98	4.95	5.02	5.52	165.72***	1 < (2 ≈ 3 ≈ 4) < 5
Adult	4.90	5.06	4.88	5.03	5.43	796.06***	(1 ≈ 3) < (2 ≈ 4) < 5
Older	5.02	4.97	4.98	5.14	5.45	104.34***	(1 ≈ 2 ≈ 3) < 4 < 5

Note. Analyses were run utilizing the BCH procedure. The values for the outcome are the means for each profile. Class numbers indicate profiles that are significantly different at least at *p* < .05. ***p < .001. class 1 = low social support class, class 2 = predominantly proximal support class, class 3 = predominantly remote support class, class 4 = moderate social support class, class 5 = high social support class

**Fig. 2:**
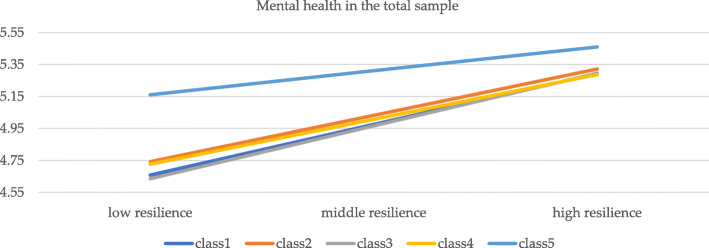
The interaction between social support and resilience on mental health in the total sample. Note. class 1 = low social support class, class 2 = predominantly proximal support class, class 3 = predominantly remote support class, class 4 = moderate social support class, class 5 = high social support class

To examine the interaction of social support and resilience on mental health across age groups, the three-way interaction was tested by Process 3.4 in the total sample. Gender and disease history were included as the control variables in model 3. We encoded the results of social support classification into dummy variables, with class 1 (low social support) as the reference category. The results are presented in Table 5, which indicated the interaction between social support and resilience on mental health was significant, and people with moderate/high (class 4/class 5) level of social support profile will buffer the negative effect of low levels of resilience on mental health during the COVID-19 pandemic (*B* = − 0.05, *p* = 0.020; *B* = − 0.24, *p* < .001). Therefore, hypothesis 3 was supported. Figure 2 shows the effect of mental health on COVID-19-related supports at high (+ 1 SD), middle, and low (− 1 SD) levels of resilience. Furthermore, although the X2*resilience*age interaction effect was significant (*B*_X2*resilience*age_ = 0.003, *p* = 0.049), the simple slope tests of social support and resilience on mental health in each age group showed that there were no significant differences in those patterns among the three age groups (*B*_*emerging adults*_ = − 0.13, *p* = 0.061; *B*_*adults*_ = 0.03, *p* = 0.117; *B*_*older adults*_ = 0.02, *p* = 0.687). Therefore, hypothesis 6 was not supported.

**Table 5:** The interaction between social support and resilience on mental health

		MHI			
		***B***	***SE***	***T***	***P***
Total sample (*N* = 23,192)	constant	5.00	0.02	239.67	<.001
	X1(class2)	0.06	0.02	3.65	<.001
	X2(class3)	−0.01	0.01	− 0.59	0.555
	X3(class4)	0.03	0.02	2.02	0.043
	X4(class5)	0.34	0.02	15.75	<.001
	resilience	0.45	0.02	30.00	<.001
	X1*resilience	−0.04	0.02	−1.66	0.096
	X2*resilience	0.02	0.02	1.06	0.288
	X3*resilience	−0.05	0.02	−2.32	0.020
	X4*resilience	−0.24	0.02	−10.05	<.001
	age	0.003	0.001	2.97	0.003
	X1*age	−0.002	0.001	−2.22	0.026
	X2*age	−0.001	0.001	−1.10	0.270
	X3*age	−0.001	0.001	−0.99	0.323
	X4*age	−0.001	0.001	−0.68	0.496
	resilience*age	−0.002	0.001	−1.57	0.116
	X1*resilience*age	0.002	0.002	1.47	0.142
	X2*resilience*age	0.003	0.001	1.97	0.049
	X3*resilience*age	0.003	0.002	1.78	0.075
	X4*resilience*age	0.000	.002	.043	.966
	gender	−0.006	0.01	−0.63	0.531
	diseases	−0.09	0.01	−6.88	<.001
	*R*^*2*^	0.000			0.153

Note. class 2 = predominantly proximal support class, class 3 = predominantly remote support class, class 4 = moderate social support class, class 5 = high social support class

## Discussion

Our primary goal in this study was to examine the main effect of sources of social support, resilience, and their interaction on the mental health of different age groups during the COVID-19 pandemic. Consistent with prior research, our study indicated that social support and resilience were the protective factors on mental health [17–19, 31, 32]. Besides, this study found some other interesting results.

First, although older adults were more likely to die after developing symptoms [62], our results showed that the mental health of older adults was not the worst among the three age groups, and was even negligibly higher than in other age cohorts, which indicates the paradox of aging [41] still exists within the context of the COVID-19 pandemic. This may be due to the older adults perceiving higher levels of family support, which could help them avoid experiencing negative emotions, according to SST [63]. Another possible reason is that our data were collected between March 25 and April 1, 2020, during which the pandemic in China was basically under control; thus, the risk of infection among older adults was significantly reduced. However, complying with social distancing guidelines may lead to lower levels of mental health in emerging adults. Additionally, for adults, facing more pressures related to life and work may lead to lower levels of mental health.

Second, there was a decline in perceived social support across age groups, except in support from family. This finding supports the notion of SST that older adults prioritize emotionally meaningful goals due to a limited time perspective, and they are more likely to spend time with emotionally close relationships than young people [44, 64]. This result is also consistent with other studies [65, 66], considering previous research on the perceived social support level across different age groups has presented conflicting results [20]. Furthermore, in this study, we used LPA to examine patterns of sources of social support in the whole sample, as well as across three age groups. Five non-parallel profiles of low social support (class 1), predominantly proximal support (class 2), predominantly remote support (class 3), moderate social support (class 4), and high social support (class 5) were identified, and the social support patterns of all groups presented consistent results. These findings highlight the importance of taking sources of support as a whole profile, rather than several independent variables, and these findings fill the gaps in the existing literature by using LPA to examine social support patterns among a large population-based sample during the COVID-19 pandemic. Notably, there were significant differences in category distribution in those groups. The distribution of emerging adults and adults in class 2 was significantly lower than that of older adults, which also provides novel evidence for SST but not in experienced staff [67].

Furthermore, the finding that the social support profiles moderate the relationship between resilience and mental health in the COVID-19 pandemic supports our compensation hypothesis, which states that social support may serve as a buffer against the impact of low levels of resilience on mental health. This result is consistent with prior research showing that the interaction effect between social support and resilience on subjective well-being is significant [8]. Moreover, age groups did not moderate the interactions between social support and resilience on mental health, as the interactions between social support and resilience on mental health showed the same patterns across age groups. For all groups, only high levels of social support (class 5) could significantly buffer mental health risks for individuals with low levels of resilience. The possible reason for this is that during the COVID-19 pandemic, psychological resilience is an essential protective factor in helping combat related mental health risks. The highest level of social support is needed to compensate for low resilience, as moderate social support or a single aspect of support cannot effectively mitigate this risk factor.

### Theoretical implications

This study has several important theoretical implications. First, to our knowledge, this is the first study to use LPA to examine patterns of sources of social support among three different age groups during the COVID-19 pandemic. Moreover, we identified five latent profiles of sources of social support: low social support (class 1), predominantly proximal support (class 2), predominantly remote support (class 3), moderate social support (class 4), and high social support (class 5). Second, the distribution of emerging adults and adults in class 2 was significantly lower than that of older adults, and there was a decline in perceived social support across age groups, except in supports from family. Therefore, these findings provide quantitative evidence for SST [42–45]. Additionally, our results also offer nuanced evidence on how social support interacts with psychological resilience and further affects mental health. Thus, high levels of social support can buffer against the negative effects of low levels of resilience on mental health.

### Practical implications

The current findings carry essential practical implications. First, according to our results, individuals should build a social support system for coping with the COVID-19 pandemic. People should keep social distancing, but not be socially isolated. During the pandemic, special attention should be paid to maintaining social connections and increasing perceived social support. Furthermore, family support is vital for all age groups, and especially for adults. The more difficulties there are to face, the more we need to provide increased social support to our family members. Individuals need to make full use of various social support resources to counteract the negative impact of the pandemic on mental health. Second, although the latent profile of sources of social support in different age groups was basically the same, there were differences in distribution, and sources of social support affected the mental health of different age groups in different patterns. Older adults perceived relatively lower social support, and only a moderate to a high level of perceived social support was found to be necessary to protect their mental health, thus reminding us that we should pay special attention to providing more social support to older adults during the pandemic. Finally, our findings suggested that high levels of resilience are necessary for young, middle-aged, and older adults to “bounce back” from the pandemic because the buffering effect of social support for different age groups was the same. When resilience is lacking, only high levels of social support resources from all aspects can compensate for the negative effects of low resilience on mental health. Therefore, it is an effective way for individuals to improve their psychological resilience against mental health risks brought on by the pandemic.

### Limitations

There are several limitations to this study. First, although our data were collected during the COVID-19 pandemic (data collection took place from March 25 to April 1, 2020), a month after the response to the major public health emergency had been lowered from level 1 to level 2 in Anhui Province. Therefore, the results may not reflect the most severe state of the sample location. Second, to reduce the difficulty of completing the questionnaires, we simplified our measurements, with each dimension of social support reflected in a single item. For instance, family support did not distinguish between support from a spouse and support from other family members, which has been found to have different effects on individual mental health in previous studies [68]. Also, we asked about support in general in the social support measurements, and a specific type of support (e.g. instrumental, emotional) should be examined in future studies to provide a more comprehensive picture of how different social support profiles affect mental health [69]. Finally, as this study had a cross-sectional design, we considered psychological resilience to be a trait indicator. However, existing studies have found that resilience may be influenced by social support as a process indicator [70]. Therefore, based on the cross-sectional data, this study was not able to reveal this relationship.

## Conclusions

Our results showed that the mental health of older adults is negligibly higher than other age groups, thus indicating that the paradox of aging still exists within the context of the COVID-19 pandemic. Furthermore, the distribution of emerging adults and adults in class 2 (relatively high family support, low other social support group) was significantly lower than that of older adults (17.3% = 17.8% < 22.6%), which provides novel evidence in support of SST. Additionally, the level of mental health of class 2 was higher than that of class 3 in adults, indicating that proximal connections (support from family) are more important for protecting mental health during an outbreak than remote support (support from other sources) in adults. Finally, our results showed that resilience is a positive predictor of mental health during the COVID-19 pandemic, and social support can serve as a buffer against the impact of low levels of resilience on mental health. However, age groups did not moderate the interaction between social support and resilience on mental health, as the interaction between social support and resilience on mental health showed the same patterns across age groups; thus, for all individuals, only high levels of social support can significantly buffer the impact of low levels of resilience on mental health.

## Data Availability

All the data supporting our findings have been presented in the manuscript; the datasets used and/or analyzed during the current study are available from the corresponding author on reasonable request.

## Abbreviations

COVID-19: Coronavirus disease 2019
LPA: Latent profile analysis
SST: Socioemotional selectivity theory
CD-RISC: Connor-Davidson Resilience Scale
MHI-5: Mental Health Inventory
AIC: Akaike information criterion
BIC: Bayesian information criterion
LMR-LRT: Lo–Mendell–Rubin likelihood ratio test
BLRT: Bootstrap likelihood ratio test
